# Diverting less urgent utilizers of emergency medical services to primary care: is it feasible? Patient and morbidity characteristics from a cross-sectional multicenter study of self-referring respiratory emergency department consulters

**DOI:** 10.1186/s13104-021-05517-8

**Published:** 2021-03-24

**Authors:** Felix Holzinger, Sarah Oslislo, Rebecca Resendiz Cantu, Martin Möckel, Christoph Heintze

**Affiliations:** 1 Institute of General Practice, Charité – Universitätsmedizin Berlin, corporate member of Freie Universität Berlin, Humboldt-Universität zu Berlin, and Berlin Institute of Health, Charitéplatz 1, 10117 Berlin, Germany; 2 Division of Emergency Medicine, Charité – Universitätsmedizin Berlin, corporate member of Freie Universität Berlin, Humboldt-Universität zu Berlin, and Berlin Institute of Health, Campus Mitte and Virchow, Berlin, Germany

**Keywords:** Emergency department, Emergency medical services, Consultation determinants, Health care utilization, Respiratory conditions

## Abstract

**Objective:**

Diversion of less urgent emergency medical services (EMS) callers to alternative primary care (PC) is much debated. Using data from the EMACROSS survey of respiratory ED patients, we aimed to characterize self-referred EMS patients, compare these with non-EMS patients, and assess scope and acceptability of a potential redirection to alternative PC.

**Results:**

Of n = 292 self-referred patients, n = 99 were transported by EMS. Compared to non-EMS patients, these were older, triaged more urgently and arrived out-of-hours more frequently. The share of chronically and severely ill patients was greater. Out-of-hours ED visit, presence of a chronic pulmonary condition as well as a hospital diagnosis of respiratory failure were identified as determinants of EMS utilization in a logistic model, while consultation for access and quality motives as well as migrant status decreased the probability. EMS-transported lower urgency outpatients visiting during regular physicians’ hours were defined as potential PC cases and evaluated descriptively (n = 9). As a third was medically complex and potentially less suitable for PC, redirection potential could be estimated at only 6% of EMS cases. This would be reduced to 2% if considering patients’ judgment concerning the appropriate setting. Overall, the scope for PC diversion of respiratory EMS patients seems limited.

## Introduction

Emergency medical services (EMS) are designated to provide pre-hospital care and transportation for very urgent and life-threatening constellations. However, EMS calls for less urgent conditions are abundant [1], and a considerable proportion of EMS patients is discharged from emergency departments (ED) after outpatient treatment [2]. From the patient perspective, EMS consultation decisions are connected with complex socioemotional factors and practical considerations, as well as subjective symptom burden [3]. The German Advisory Council on the Assessment of Developments in the Health Care Sector recently proposed optionally diverting less urgent cases to primary care (PC) [4]. Currently, there is no redirection pathway in Germany, neither on initiative of EMS dispatch or EMS personnel; calls usually result in ambulance deployment and transport to hospital. However, there is insufficient data on how urgent and severely ill EMS callers are and concerning the scope for redirection. A US study suggests that up to a third of EMS calls resulting in ED outpatient treatment could have been managed in non-hospital settings [5]. For the UK, redirection potential has been estimated at 12% [6], and at comparable 16% in a Swedish study [7]. However, estimates rely on secondary and retrospective data or expert opinions. The mixed-methods EMACROSS (Emergency and Acute Care for Respiratory Diseases beyond Sectoral Separation) study investigated ED patients with respiratory complaints. Such are a good model to study ED utilization and interactions with PC, constituting frequent consultation triggers in both settings [8, 9] and comprising a wide causal spectrum, from medically banal to serious. Cross-sectional data from EMACROSS showed lesser acute and chronic morbidity in self-referred walk-in ED patients [10]. In extension of this preceding publication, the present paper provides an in-depth look at self-referred EMS patients—defined as cases in which the decision to call EMS was the patients’ own, rather than a referring physician’s—to evaluate the potential for PC as an alternative option.

## Main text

### Methods

#### Study design and data collection

Setting, design and data collection of EMACROSS, subproject of EMANet (Emergency and Acute Medicine Network for Health Care Research) [11], have been described previously [10]. We collected primary (patient survey) and secondary (hospital records) data on respiratory ED consultations, complemented by qualitative patient and provider interviews. Patients presenting with respiratory symptoms (e.g. dyspnea, cough) were recruited in eight EDs in Berlin’s central district (Berlin-Mitte). Patients > 18 years were eligible if able to give written informed consent and proficient in one of the study languages. Participants were recruited between 1st of June 2017 and 30th of November 2018 and surveyed at ED presentation prior to being seen, or between investigations, with a tablet-based questionnaire comprising demographic and medical characteristics, consultation motives and health care utilization (see supplement to [10]). We also refer to this previous publication for details on operationalization of consultation motives, which were assessed as multi-response data and appropriated to thematic groups (distress, access, quality, and convenience). In the analyses presented here, only self-referred patients (EMS alerted on patient’s initiative) were included, redirection to PC not being a realistic option in referred cases (necessity of the ED pre-determined by a health professional). Among EMS consulters, potential PC diversion cases were filtered by criteria of triage category (less urgent, Manchester Triage System levels 4 and 5), presentation time (during usual office hours, see legend to Table 1), and management as an outpatient, as only such cases were deemed to represent the real redirection potential. Patients’ subjective judgment regarding PC as an alternative (patients were asked whether a GP could also have solved their problem) was assessed as indicator of potential acceptance of diversion.

**Table 1:** Characteristics of self-referred study participants and EMS vs. non-EMS subgroups

Variable	Measure	Group			p value for group difference
		Total	EMS	non-EMS	EMS vs. non-EMS
Participants	n	292	99	188	
*Demographics*					
Age	n	292	99	188	
	Mean (SD) Median (Range)	51.34 (18.89) 51.5 (18–96)	62.56 (16.33) 66.0 (19–92)	45.34 (17.46) 43.0 (18–96)	< 0.001
Sex	n	292	99	188	
Male	%	51.4	57.6	47.3	0.10
Female	%	48.6	42.4	52.7	
Migration and travel	n	291	98	188	
Migrant first generation	%	26.8	11.2	35.1	< 0.001
Second generation	%	7.6	5.1	8.5	
Tourist	%	6.5	3.1	8.5	
Education (CASMIN)	n	289	98	186	
Low	%	23.2	36.7	16.1	< 0.001
Intermediate	%	41.5	42.9	39.8	
High	%	35.3	20.4	44.1	
*ED consultation*					
Triage category	n	284	96	183	
Lower urgency	%	43.3	22.9	54.1	< 0.001
Higher urgency	%	56.7	77.1	45.9	
Time of presentation	n	292	99	188	
Out-of-hours visit	%	23.6	33.3	18.1	0.004
During office hours	%	76.4	66.7	81.9	
Symptom-associated distress	n	277	93	180	
	Mean (SD) Median (Range)	7.29 (1.78) 7.5 (1.5–10)	7.95 (1.64) 8.5 (2.0–10)	6.96 (1.78) 7.0 (1.5–10)	< 0.001
*Chronic conditions and care*					
Chronic pulmonary condition	n	291	99	187	
	%	57.7	81.8	44.4	< 0.001
Multimorbidity	n	290	99	186	
	%	52.1	74.7	40.3	< 0.001
Attached to GP	n	290	99	186	
	%	82.4	92.9	76.9	< 0.001
*Mental health*					
PHQ4 score	n	291	98	188	
	Mean (SD) Median (Range)	4.03 (3.62) 3.0 (0–12)	3.96 (3.72) 3.0 (0–12)	4.07 (3.61) 3.0 (0–12)	0.69
*ED visit outcomes*					
Diagnoses	n	292	99	188	
Pneumonia J12-J18	%	16.8	24.2	12.2	0.009
COPD and chronic bronchitis J40-J44	%	32.9	54.5	20.7	< 0.001
Asthma bronchiale J45-J46	%	12.0	7.1	14.4	0.07
Other respiratory tract infection J09-J11, J20-J22	%	9.6	8.1	10.6	0.49
Upper airway conditions J0x/J3x	%	11.6	4.0	16.0	0.003
Respiratory symptom diagnosis only (R section code)	%	14.7	10.1	17.6	0.09
Respiratory failure J96	%	17.8	36.4	7.4	< 0.001
Visit consequence	n	292	99	188	
Outpatients	%	62.0	35.4	76.6	< 0.001
Hospital admission	%	38.0	64.6	23.4	
*Consultation motive categories*	n	292	99	188	
Distress	%	69.9	74.7	66.5	0.20
Access	%	30.8	17.2	37.8	< 0.001
Quality	%	4.1	9.1	23.9	0.002
Convenience	%	18.5	2.0	5.3	0.18
*Potential primary care cases*	n	284	96	183	
Meeting criteria	%	29.6	9.4	40.1	< 0.001

n = cases with available data for respective characteristic; % = percentage of cases with available data; Means of arrival unknown for n = 5 participants, thus not categorized into EMS/non-EMS; Migration and travel: first generation = not born in Germany, second generation = born in Germany and mother/father (or both) born in another country; Time of presentation: out-of-hours defined as between 6 pm and 8 am on weekdays, plus weekends and Wednesdays afternoons after 2 pm; Subjective symptom-associated distress: 0–10 scale; PHQ4: 0–12 scale; Chronic pulmonary condition: if either self-reported or documented in hospital records; Multimorbidity: two or more chronic conditions reported by patient; Diagnoses: ICD-10 codes, multiple diagnoses possible for individual cases.; Motive categories: multi-response data, n = 265 (90.8%) attested to one or more of the four motive categories, percentages in table refer to total of cohort/group; “Distress” motive: symptom severity and anxiety; “Access”: service–defined barriers to alternative care; “Quality”: expectations of better care in hospital; “Convenience”: comfort and ease of ED access; Statistical comparisons of groups: χ2 test for categorical and Mann–Whitney-U-test for continuous variables

#### Data analysis

For allocation of self-referral status, we used available data from both survey and hospital records; EMS utilization was determined from ED documentation. For details on data preparation, see [10]. Demographics, morbidity characteristics, and motives of EMS vs. non-EMS patients were summarized descriptively, and group comparisons were performed by χ^2^ test for categorical and Mann–Whitney-U-test for continuous variables. Predictors of EMS consultations were determined by binary logistic regression. Based on the literature, we compiled a set of variables of interest as potential predictors or control variables. We carried out univariate statistics; non-significant variables were retained if potentially important (e.g. as control variables, or if discussed as relevant by others) [12]. A preliminary multivariate model was constructed, effects of discarding variables were checked. Candidate models were compared as to fit and predictive accuracy by R2, Hosmer Lemeshow test, and AUC (area under the ROC curve) [13]. We refrained from stepwise selection to avoid associated bias. Potential PC diversion cases are presented descriptively as a small case series.

### Results

Of n = 472 participants in the EMACROSS cohort, n = 292 (61.9%) were self-referrals. Of these, n = 99 arrived in the ED via EMS transport. Table 1 shows characteristics of all self-referred cases and comparisons of EMS vs. non-EMS patients. The EMS group showed higher age, contained a greater proportion of male patients and a lesser share of first-generation migrants. EMS patients were triaged in more urgent categories and arrived out-of-hours more frequently. The share of chronically ill (both pulmonary and otherwise) patients was also greater, with markedly larger proportions of COPD and diagnoses of respiratory failure. Hospital admission was considerably more frequent in the EMS group.

In the logistic model, which showed good predictive ability at an AUC of 0.87, higher age, an out-of-hours ED visit, and presence of a chronic pulmonary condition as well as a hospital diagnosis of respiratory failure were identified as determinants of EMS utilization. In contrast, reporting “access” and “quality” consultation motives decreased the probability of a visit via EMS transportation, as well as being a first-generation migrant (Table 2).

**Table 2:** Logistic regression model for EMS vs. non-EMS transport as dependent variable (n = 268 complete cases)

Independent variable	Coefficient B	Standard error	p value	Odds ratio	OR 95% CI lower bound	OR 95% CI upper bound
Age	0.04	0.01	< 0.001	1.04	1.02	1.06
Sex *Reference: female*	0.08	0.34	0.82	1.08	0.55	2.12
Migration and travel *Reference: no related feature*						
Migrant first generation	− 0.97	0.45	0.03	0.38	0.16	0.91
Second generation	− 0.22	0.74	0.76	0.80	0.19	3.40
Tourist	− 0.83	0.76	0.28	0.44	0.10	1.95
Triage category *Reference: lower urgency*	0.43	0.37	0.24	1.54	0.75	3.18
Out-of-hours visit	0.90	0.40	0.02	2.45	1.12	5.34
Chronic pulmonary condition	1.51	0.38	< 0.001	4.53	2.15	9.55
Respiratory failure diagnosis	1.02	0.43	0.02	2.77	1.18	6.47
Consultation motive “access”	− 1.13	0.40	0.00	0.32	0.15	0.70
Consultation motive “quality”	− 1.21	0.52	0.02	0.30	0.11	0.82

Model performance metrics (for model containing all above variables): AUC 0.87; Nagelkerke R^2^ 0.50; Hosmer–Lemeshow test χ^2^ = 5.142, df = 8, p = 0.742

To get more information concerning the scope for redirection of less urgent cases to PC, we filtered potential PC cases by the criteria outlined above. Only nine cases (9.4%) met this definition. A detailed evaluation of these patients was conducted to determine their potential eligibility for alternative PC care. Table 3 presents characteristics of this small case series. Data is reported descriptively due to the small subsample.

**Table 3:** Details of potential PC cases (n = 9) among EMS patients (outpatients triaged of lower urgency visiting during regular physicians’ hours)

Attribute	Measure(s)	Value(s)	Comments
Age	Mean (SD) Median (Range)	49.33 (24.51) 53.0 (19–77)	Four patients were > 65 years of age
Sex	n		
Male		4	
Female		5	
Migration and travel	n		
Migrant first generation		1	Has lived in Germany for > 40 years
Tourist		1	Born in Germany, but lives abroad
Education	n		
Low		2	
Intermediate		5	
High		2	
MTS category	n		
4		8	None had abnormal respiratory rate or a fever
5		1	
First-time symptoms	n	3	
Symptom onset	n		
Same day		4	Presenting symptoms (multiple indications allowed) were cough n = 2, dyspnea n = 7, thoracic pain n = 1, fatigue = 1, dizziness n = 1
Longer		5	
Symptom-associated distress	Mean (SD) Median (Range)	8.33 (1.32) 8.5 (5.5–10.0)	Symptom severity (as component of distress) was rated as 10 by n = 2 and 9 by n = 3 on a 0–10 scale
ED Diagnoses	case no		
	1	R05	Cough
	2	J06.9	RTI
	3	J20.9 R09.1	Acute bronchitis Pleuritis
	4	M31.3 no code given	Wegener’s Granulomatosis Gastroenteritis
	5	E86	Dehydration
	6	R06.0	Dyspnea
	7	R06.0	Dyspnea
	8	R11	Nausea and vomiting
	9	no code given	Psychogenic dyspnea
Chronic pulmonary condition	n	6	Other notable chronic conditions: HIV n = 1 Lung cancer n = 1 Wegener’s granulomatosis (incl. kidney disease with peritoneal dialysis) n = 1 Implanted defibrillator after resuscitation due to ventricular fibrillation n = 1
Multimorbidity	n	5	
Attached to GP	n	9	Six reported to have been a patient there for five years or longer, all reported to be either satisfied or very satisfied (5-point likert scale) with their GP
Other ED visit(s) in past six months	n	4	Four patients reported an inpatient stay, three of these also had an ED visit
PHQ4 score	Mean (SD) Median (Range)	4.67 (3.94) 4.00 (0–11)	Two cases with score ≥ 9
Consultation motive categories	n		None reported in “quality” and “convenience” categories
Distress		6	
Access		1	

Subjective symptom-associated distress: 0–10 scale; PHQ4: 0–12 scale; Chronic pulmonary condition: if either self-reported or documented in hospital records; Multimorbidity: two or more chronic conditions reported by patient; Diagnoses: ICD-10 codes (if documented in ED) or free-text diagnoses, depending on manner of documentation; Motive categories: multi-response data

In this subsample, data suggests a discrepancy between low-urgency triage and patients’ subjective judgment of the situation. Although all patients had normal respiratory rates and none had respiratory failure, subjective distress was high. Most cases in this small subsample had a chronic pulmonary condition, while—corresponding to their management as outpatients—ED diagnoses do not suggest a clinically critical situation. However, other serious chronic illnesses indicating complex health problems (lung cancer, HIV, Wegener’s granulomatosis with kidney failure) were present in a third, and a considerable share had been hospitalized in preceding months. Three of the four formerly hospitalized patients reported an inpatient stay of more than 30 days duration. Considering potential acceptability of a diversion to PC, only two of nine believed a GP could also have solved their acute problem.

### Discussion

In this respiratory cohort, EMS utilization—compared to other means of ED access—is associated with “sicker” patients, as indicated by e.g. higher acute and chronic pulmonary morbidity. While there exist few comparisons of EMS vs. non-EMS patients, similar findings have been reported for e.g. patients presenting with chest pain [14]. The association of EMS utilization with age [15–17] as well as frailty [18] has likewise been described. Male gender did not constitute an independent EMS utilization predictor in our cohort, as opposed to other studies [16, 19] not limited to respiratory patients. Our data shows high proportions of consultations during regular office hours in both EMS and non-EMS cases, corresponding to the literature on temporal patterns of emergency services demand [20]. The higher chance of EMS cases consulting out-of-hours seems plausible: people may be more inclined to call EMS dispatch e.g. at night, with alternative transport more difficult to attain. Concerning appropriateness of EMS calls, utilization in our sample seems predominantly as intended, data not indicating prevalent irresponsible use of ED via EMS as an easy PC alternative, even if such would be assumed for all cases meeting our potential PC patient definition. This is supported by the greater importance of “access” and “quality” consultation motives in non-EMS patients: practice holidays or waiting times for PC appointments as well as considerations of getting optimal care in hospital do prompt a share of ED consultations, but these consulters do not seem to call on EMS usually. Comparing consultation determinants identified in our data with other investigations, it strikes that first-generation migrants appear less likely to call on EMS. Correspondingly, a lower tendency to utilize pre-hospital emergency care in first-generation migrants—as compared to second-generation migration background—has been reported from a representative survey of German consulters [21], but the authors could not explain this difference. Conceivable underlying factors could encompass barriers to making the call to dispatch—which has been discussed in the context of refugees [22]—as well as a tendency to relying on e.g. family members for transport, but this remains speculative. In this context, a large observational study from Denmark had identified living alone as a determinant of utilizing EMS, besides age, low income and receipt of cash benefits [17].

As to the potential for redirecting some EMS patients to PC, our detailed look at less-urgent outpatient cases consulting during regular office hours suggests that some of these feature complex long-term health problems. Combined with an acute respiratory distress situation, ED care might be more appropriate than PC here: a GP might have referred to hospital anyway. The actual diversion potential in our cohort thus amounts to about 6% of cases. However, the high level of symptom-associated distress in this small cluster of potential PC patients indicates that the patient perspective may differ fundamentally, patients potentially considering themselves real emergencies requiring ED care. Acceptance of diversion would thus supposedly be low, and total redirection potential would be reduced to only 2% if considering patients’ affirmation of being managed by a GP. This falls markedly below other estimates of diversion potential [5–7], mainly due to their different—and in our view less realistic—selection criteria. A principal shortcoming of all retrospective classifications of appropriate ED cases vs. potential PC patients is the imminent neglect of the acceptability of a conceivable diversion, data not indicating whether patients would embrace or refuse such. Patient support for alternative care is essential for any successful redirection scheme, and this was low in our study, as e.g. compared to an unselected ED sample [23]. Another important issue is the accuracy and safety of paramedic decisions regarding necessity of ED care, for which evidence is not clear [24]. Overall, this investigation casts a measure of doubt on the expediency of efforts to redirect EMS consulters with respiratory symptoms to PC, as the status quo of utilization seems predominantly appropriate and realistic diversion potential is small.

## Limitations

Several limitations apply. Most importantly, our cohort consists of patients with respiratory complaints, and symptoms like dyspnea are potentially less straightforward to judge for both patients and health professionals—including dispatch operators and EMS personnel—than e.g. a minor injury. Contemplating PC diversion may be comparably complicated in respiratory cases, as it may become apparent only after investigations that the situation is non-serious. This limits generalizability. Secondly, the small group of potential PC cases selected by realistic, but comparably restrictive criteria, does not allow for inferential analyses. However, it illustrates the diversity of cases and the difficulty of judging eligibility for one care sector vs. another. Thirdly, the documented share of out-of-hours consultations was potentially influenced by recruitment times, study personnel being present only intermittently off-hours. Speculatively, EMS patients could also be comparably less inclined to consent to study participation, especially at night. Lastly, as data was collected in EDs, we do not know how patients initially presented to EMS personnel. It would be important to learn about their judgment regarding the level of care required, because the redirection decision would lie with EMS. To address these limitations, a follow-up study on the specific question of PC redirection potential considering EMS data and perspective is in preparation.

## Data Availability

The datasets used and analyzed during this study are available from the corresponding author on reasonable request.

## Abbreviations

EMS: Emergency medical services
ED: Emergency department
PC: Primary care
US: United States
UK: United Kingdom
ROC: Receiver operating characteristic
AUC: Area under the curve
MTS: Manchester Triage System
GP: General practitioner
COPD: Chronic obstructive pulmonary disease
CASMIN: Comparative Analysis of Social Mobility in Industrial Nations
PHQ: Patient Health Questionnaire
ICD: International Classification of Diseases
HIV: Human immunodeficiency virus

## References

[1] Booker MJ, Purdy S, Barnes R, Shaw ARG (2019). Ambulance use for ‘primary care’ problems: an ethnographic study of seeking and providing help in a UK ambulance service. BMJ Open.

[2] Hegenberg K, Trentzsch H, Prückner S (2019). Differences between cases admitted to hospital and discharged from the emergency department after emergency medical services transport. BMJ Open.

[3] Booker MJ, Purdy S, Shaw ARG (2017). Seeking ambulance treatment for ‘primary care’ problems: a qualitative systematic review of patient, carer and professional perspectives. BMJ Open.

[4] Advisory Council on the Assessment of Developments in the Health Care System. Report 2018: Needs-Based Regulation of the Health Care Provision. Berlin: Deutscher Bundestag 19. Wahlperiode; Drucksache 19/3180 [Full-length version; German language]; 2018.

[5] Alpert A, Morganti KG, Margolis GS, Wasserman J, Kellermann AL (2013). Giving EMS flexibility in transporting low-acuity patients could generate substantial medicare savings. Health Aff.

[6] Woollard M (2003). Emergency calls not requiring an urgent ambulance response: expert consensus. Prehosp Emerg Care.

[7] Norberg G, Wireklint Sundström B, Christensson L, Nyström M, Herlitz J (2015). Swedish emergency medical services’ identification of potential candidates for primary healthcare: Retrospective patient record study. Scand J Prim Health Care.

[8] Backman AS, Blomqvist P, Lagerlund M, Carlsson-Holm E, Adami J (2008). Characteristics of non-urgent patients. Cross-sectional study of emergency department and primary care patients. Scand J Prim Health Care..

[9] Silva DR, Viana VP, Müller AM, Coelho AC, Deponti GN, Livi FP (2013). Epidemiological aspects of respiratory symptoms treated in the emergency room of a tertiary care hospital. Jornal brasileiro de pneumologia : publicacao oficial da Sociedade Brasileira de Pneumologia e Tisilogia.

[10] Holzinger F, Oslislo S, Möckel M, Schenk L, Pigorsch M, Heintze C (2020). Self-referred walk-in patients in the emergency department—who and why? Consultation determinants in a multicenter study of respiratory patients in Berlin, Germany. BMC Health Serv Res.

[11] Schmiedhofer M, Inhoff T, Krobisch V, Schenk L, Rose M, Holzinger F (2018). EMANet: a regional network for health services research in emergency and acute medicine. Z Evid Fortbild Qual Gesundhwes.

[12] Heinze G, Wallisch C, Dunkler D (2018). Variable selection—a review and recommendations for the practicing statistician. Biom J.

[13] Stoltzfus JC (2011). Logistic regression: a brief primer. Acad Emerg Med.

[14] Nowak B, Giannitsis E, Riemer T, Münzel T, Haude M, Maier LS (2012). Self-referral to chest pain units: results of the German CPU-registry. Eur Heart J Acute Cardiovasc Care.

[15] Shah MN, Glushak C, Karrison TG, Mulliken R, Walter J, Friedmann PD (2003). Predictors of emergency medical services utilization by elders. Acad Emerg Med.

[16] Clark MJ, FitzGerald G (1999). Older people's use of ambulance services: a population based analysis. J Accid Emerg Med.

[17] Søvsø MB, Bech BH, Christensen HC, Huibers L, Christensen EF, Christensen MB (2020). Sociodemographic characteristics associated with contacts to emergency medical services and out-of-hours primary care: an observational study of 2.3 million citizens. Clin Epidemiol..

[18] Vinson AJ, Bartolacci J, Goldstein J, Swain J, Clark D, Tennankore KK (2020). Predictors of need for first and recurrent emergency medical service transport to emergency department after dialysis initiation. Prehosp Emerg Care..

[19] Tangherlini N, Pletcher MJ, Covec MA, Brown JF (2010). Frequent use of emergency medical services by the elderly: a case-control study using paramedic records. Prehosp Disaster Med.

[20] Cantwell K, Dietze P, Morgans AE, Smith K (2013). Ambulance demand: random events or predicable patterns?. Emerg Med J.

[21] Kietzmann D, Knuth D, Schmidt S (2017). (Non-)utilization of pre-hospital emergency care by migrants and non-migrants in Germany. Int J Public Health.

[22] Sheikh M, Nugus P, Gao Z, Holdgate A, Short A, Alhaboub A (2011). Equity and access: understanding emergency health service use by newly arrived refugees. Med J Aust.

[23] Munjal KG, Shastry S, Loo GT, Reid D, Grudzen C, Shah MN (2016). Patient perspectives on EMS alternate destination models. Prehosp Emerg Care.

[24] Fraess-Phillips AJ (2016). Can paramedics safely refuse transport of non-urgent patients?. Prehosp Disaster Med.

